# The Relationship between C-Reactive Protein Level and Discharge Outcome in Patients with Acute Ischemic Stroke

**DOI:** 10.3390/ijerph13070636

**Published:** 2016-06-27

**Authors:** He-Hong Geng, Xin-Wang Wang, Rong-Li Fu, Meng-Juan Jing, Ling-Ling Huang, Qing Zhang, Xiao-Xiao Wang, Pei-Xi Wang

**Affiliations:** 1 Institute of Public Health, School of Nursing, Henan University, Kaifeng 475004, China; hehong0409@163.com (H.-H.G.); jing53905@163.com (M.-J.J.); huanglingling0703@163.com (L.-L.H.); zq71200@163.com (Q.Z.); xiaoxiao52625@163.com (X.-X.W.); 2 Department of Preventive Medicine, School of Public Health, Guangzhou Medical University, Guangzhou 510182, China; xinwwang@aliyun.com; 3 Department of Neurology of Huai-He Hospital, Kaifeng 475000, China; 15225475563@163.com

**Keywords:** acute ischemic stroke, C-reactive protein, recurrence, discharge outcome

## Abstract

Previous studies showed that C-reactive protein (CRP), an inflammatory marker, was associated with stroke severity and long-term outcome. However, the relationship between the acute-phase CRP level and discharge outcome has received little attention. We prospectively studied 301 patients with acute ischemic stroke (over a period of two weeks) from two hospital stroke wards and one rehabilitation department in Henan, China. Patients’ demographic and clinical data were collected and evaluated at admission. Poor discharge outcome was assessed in patients at discharge using the Modified Rankin Scale (MRS > 2). Multivariate logistic regression analysis was performed to determine the risk factors of poor discharge outcome after adjusting for potential confounders. Poor discharge outcome was observed in 78 patients (25.9%). Univariate analyses showed that factors significantly influencing poor discharge outcome were age, residence, recurrent acute ischemic stroke, coronary heart disease, the National Institutes of Health Stroke Scale (NIHSS) score at admission, non-lacunar stroke, time from onset of stroke to admission, CRP, TBIL (total bilirubin), direct bilirubin (DBIL), ALB (albumin), FIB (fibrinogen) and D-dimer (*p* < 0.05). After adjusting for age, residence, recurrent ischemic stroke, coronary heart disease, NIHSS score at admission, lacunar stroke, time from onset of stroke to admission, CRP, TBIL, DBIL, ALB, FIB and D-dimer, multivariate logistic regression analyses revealed that poor outcome at discharge was associated with recurrent acute ischemic stroke (OR, 2.115; 95% CI, 1.094–4.087), non-lacunar stroke (OR, 2.943; 95% CI, 1.436–6.032), DBIL (OR, 1.795; 95% CI, 1.311–2.458), and CRP (OR, 4.890; 95% CI, 3.063–7.808). In conclusion, the CRP level measured at admission was found to be an independent predictor of poor outcome at discharge. Recurrent acute ischemic stroke, non-lacunar stroke and DBIL were also significantly associated with discharge outcome in acute ischemic stroke.

## 1. Introduction

Stroke is regarded not only as the most devastating neurological disease, often resulting in death or physical impairment and disability [1], but it also is the second most common cause of death and the leading cause of adult disability in China [2]. Ischemic stroke (IS), also called cerebral infarction, is the most common type of stroke and accounts for about 80%–85% of stroke cases [3]. Over the last few decades, inflammation has been proposed to play an important role in the pathogenesis of acute ischemic stroke (AIS) [4,5,6,7,8,9,10]. C-reactive protein (CRP), which is the classical acute phase reactant protein, is viewed as the most extensively studied marker of inflammation [11,12,13,14,15]. Undoubtedly, it is also one of the most widely studied inflammatory biomarkers in cardiovascular disease and IS [16,17,18,19,20].

In recent years, an increased level of CRP remarkably associated with the functional prognosis of AIS was observed in multiple studies [4,21,22,23,24,25,26,27]. Nevertheless, most of the previous studies investigating the prognosis of patients with acute ischemic stroke were mainly focused on new stroke attack and mortality [21,23,24,28,29,30,31,32,33]. In addition, Halvor et al. [33] found that CRP and homocysteine were associated with long-term mortality in young ischemic stroke patients. Huang et al. [30] revealed that hs-CRP was related to a worse prognosis risk of all-cause death within three months after AIS in Chinese patients. Furthermore, Whiteley et al. [16] summarized the relationship between CRP, homocysteine, cholesterol, and fibrinogen in the blood sample and the functional prognosis of AIS. At present, it is uncertain whether other factors, such as total bilirubin (TBIL), direct bilirubin (DBIL) and D-dimer, may affect the risk of discharge outcome with AIS. The majority of the studies concentrated on the time of outcome of more than three months [24,25,30,31,34,35,36,37], but few studies have analyzed the relationship between the CRP level at admission and the discharge outcome. It is therefore necessary to study the relationship between the CRP level at admission and the discharge outcome and identify the possible risk factors of discharge outcome with AIS for developing effective preventive measures.

## 2. Methods

### 2.1. Study Population

**Figure 1:**
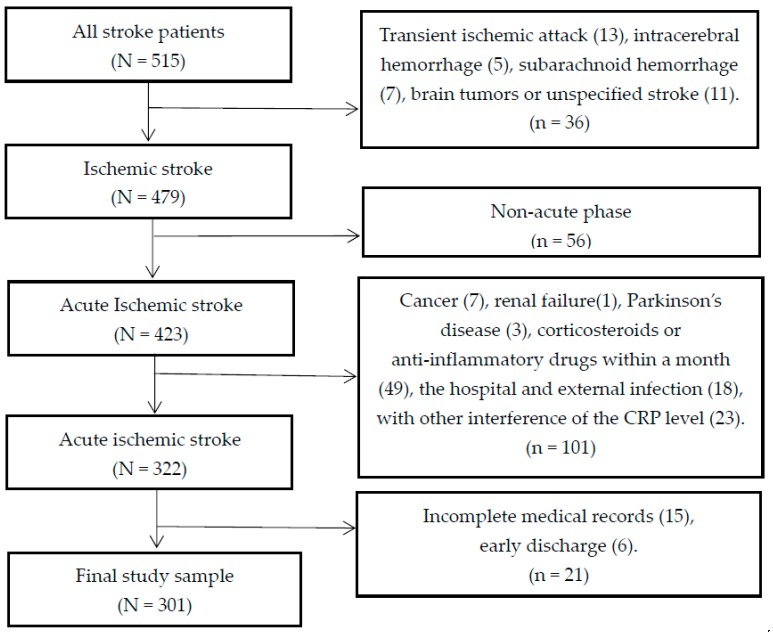
Flow chart in the selection of study patients.

In this longitudinal study, we recruited a total of 515 subjects who were admitted to three stroke units (two from the Department of Neurology and one from the Department of Rehabilitation) of Huai-He Hospital, Kaifeng, China, between 30 June 2015 and 30 November 2015. All patients with first-ever or recurrent acute ischemic stroke (a history of stroke) during the acute phase (symptoms occurring within 14 days [30]) were diagnosed by a neurologist according to the fourth Chinese National Conference’s recommendations on the diagnosis of cerebrovascular diseases [38]. All potentially eligible patients were screened. The inclusion criteria were age of 18 years or older; willingness to provide informed consent; and accepting the same conservative treatment. The study exclusion criteria included diagnosis with transient ischemic attack (TIA), intracerebral hemorrhage (ICH), subarachnoid hemorrhage (SAH), brain tumors or unspecified stroke; a history of more serious medical disease other than ischemic stroke such as cancer, renal failure, and Parkinson’s disease; treatment with corticosteroids or anti-inflammatory drugs (such as non-specific airway responsiveness, non-steroidal anti-inflammatory drugs and statins) within a month before stroke; incomplete medical records and early discharge from the hospital (within three or fewer days) [39]; the hospital and external infection; and interference of the CRP level by any disorder, including asthma, arthritis, liver disease, bronchitis, sinus infection, pneumonia, and gout flare or arthritis flare in the past two weeks [15]. A flowchart illustrating the selection of study patients is presented in Figure 1. Finally, 301 subjects were included in this study.

### 2.2. Study Procedure

Informed consent, data about sociodemographics, prevalent medical history, risk factors for stroke, and blood samples were obtained for all patients.

#### 2.2.1. Sociodemographic Data and Lifestyle Variables

Demographic variables included age, gender, working state, marital status, residence (urban or rural), and lifestyle before stroke (including smoking and alcohol drinking). Working status was divided into working and not working (including retired and unemployed). Marital status was divided into single (including unmarried, divorced and widowed) and married. Smokers were defined as those who smoked one or more cigarettes per day for at least six months. Alcohol drinkers were defined as those who drank alcohol on average more than once per week over the past year.

#### 2.2.2. Possible Stroke-Related Clinical Parameters

Clinical parameters included the time from the onset of stroke to admission, first-ever or recurrent acute ischemic stroke, comorbid diseases (including hypertension, diabetes mellitus, and coronary heart disease), National Institute of Health Stroke Scale (NIHSS) score at admission, stroke subtypes (lacunar stroke and non-lacunar stroke), hospital day (HOD), and the Modified Rankin Scale (MRS) score at discharge. Hypertension was defined as self-reported history of hypertension and/or at least two blood pressure recordings exceeding 140/90 mmHg after admission. Diabetes mellitus (DM) was defined as self-reported history of DM and/or a fasting glucose level ≥7.0 mmol/L or random blood glucose ≥11 mmol/L in more than two separate measurements. Coronary heart disease was documented by previous history of myocardial infarction >6 months but <5 years before enrollment in the study, or angina pectoris confirmed by positive coronary angiography, nuclear scintigraphy, or exercise test [40]. Neurological stroke severity was assessed using the NIHSS within the first 24 h after admission [41,42], and was classified mild to moderate (NIHSS ≤ 15), or severe (NIHSS > 15) [32,43]. Stroke subtypes were categorized into lacunar (infarct diameter ≤ 1.5 cm) and non-lacunar (infarct diameter > 1.5 cm) [7,44,45].

#### 2.2.3. Outcome Measurement

Experts in the Department of Neurology assessed the outcome using the MRS [46] at the time of discharge from the hospital (the routine in-hospital treatment course for stroke is about 14 days). The MRS defines six different grades of disability, from 0 for “no symptoms at all” to 5 for “severe disability or bedridden, incontinent, and requiring constant nursing care and attention”, and grade 6 for death [47]. The MRS score of 0–2 was defined as good outcome (no symptoms/slight disability), while a score of 3–6 was defined as poor outcome (moderate/severe disability/death) [48,49,50].

#### 2.2.4. Blood Sampling

Blood samples were collected from the antecubital vein into tubes with EDTA (Ethylene Diamine Tetraacetic Acid) within the first 24 h after hospital admission and during fasting. All recruited patients accepted routine blood analysis for the measurement of white blood cell (WBC) counts, red blood cell (RBC) counts, hemoglobin (HGB), and biochemical factors including erythrocyte sedimentation rate (ESR), total bilirubin (TBIL), direct bilirubin (DBIL), albumin (ALB), total cholesterol (CHOL), triglyceride (TG), high density lipoprotein (HDL), low density lipoprotein (LDL), glucose (GLU), and homocysteine (HCY); and blood coagulation including fibrinogen (FIB) and D-dimer.

#### 2.2.5. CRP Assay

The serum was refrigerated at −70 °C after centrifugation at 3000 rpm for 10 min within an hour of collection and used in scattering turbidimetry and BN-100 special protein instrument test for determining the CRP level.

### 2.3. Ethics Statement

All subjects gave their informed consent for inclusion before they participated in the study. The study was conducted in accordance with the Declaration of Helsinki, and the protocol was approved by the Ethics Committee of Henan University, China (HDGW-2014-02).

### 2.4. Data Analysis

Data were analyzed using descriptive statistics (mean and standard deviation frequency, and percentage) for continuous and categorical variables, respectively. We examined the differences in continuous variables between patients with good and poor discharge outcome using student’s *t*-test for normally distributed data, and non-parametric test for non-normally distributed data. Data were compared using Pearson’s chi-square test for categorical variables. Multivariate logistic regression models were employed to investigate the risk factors of discharge outcome with a forward stepwise selection strategy. Parsimonious final logistic regression model was fitted by retaining all predictor variables with *p* < 0.05. Odds ratios (OR) with 95% confidence intervals (CI) were presented. Two-tailed *p*-values < 0.05 were considered statistically significant. All statistical analyses were performed using the SPSS software (version 17.0; Inc., Chicago, IL, USA).

## 3. Results

### 3.1. Demographic Characteristics

The baseline demographic characteristics of acute ischemic stroke patients with good and poor outcomes of discharge are presented in Table 1. Out of the 301 patients with acute ischemic stroke included in the study, 223 (74.1%) patients reported a good outcome, and 78 (25.9%) patients reported a poor outcome at discharge. The age of the patients was 33–87 years (mean of 64.13 ± 10.53 years). Male patients constituted 57.1% of the study population. The majority of participants were married (91.4%), and lived in urban areas (52.8%). The proportions of working, smoking, and alcohol drinking before stroke were 31.2%, 38.2% and 20.6%, respectively. There were no significant differences in sex, working, marital status, smoking and alcohol consumption between good and poor outcome (*p* > 0.05). However, age was significantly associated with the discharge outcome (*p* = 0.003). Additionally, residence in rural regions was associated with an increased risk of poor discharge outcome (*p* = 0.007).

### 3.2. Clinical Risk Factors and Blood Biochemical Indexes for Discharge Outcome

The comparison of clinical variables in acute ischemic stroke patients with good or poor outcome of discharge is shown in Table 2. Poor discharge outcome was not associated with HOD, hypertension, or DM, but was significantly related to recurrent acute ischemic stroke (*p* < 0.001), NIHSS score at admission (*p* = 0.001), non-lacunar stroke (*p* = 0.006), coronary heart disease (*p* = 0.044), and time from the onset of stroke to admission (*p* = 0.042). Compared to patients with good outcome, patients with poor outcome had higher CRP levels (18.86 ± 19.05 vs. 4.97 ± 6.35 mg/L, *p* < 0.001), higher TBIL levels (15.41 ± 5.75 vs. 13.51 ± 5.22 umol/L, *p* = 0.007), higher DBIL levels (5.34 ± 1.78 vs. 4.25 ± 1.81 umol/L, *p* < 0.001), lower ALB levels (38.45 ± 7.04 vs. 40.17 ± 2.91 g/L, *p* < 0.001), higher FIB levels (346.60 ± 91.46 vs. 307.41 ± 81.53 mg/dL, *p* < 0.001), and higher D-dimer levels (930.35 ± 954.95 vs. 442.40 ± 534.64 ng/mL, *p* < 0.001) at discharge, as demonstrated in Table 3. The other biochemical indexes did not differ significantly between the two groups.

The average CRP level of the study subjects was 8.57 mg/L (SD, 12.65; range, 0.00–99.99). The CRP level was weakly associated with age (*p* = 0.001; Spearman correlation coefficient, 0.197); WBC (*p* < 0.001; Spearman correlation coefficient, 0.258); DBIL (*p* = 0.006; Spearman correlation coefficient, 0.159); HDL (*p* = 0.013; Spearman correlation coefficient, −0.144); GLU (*p* = 0.004; Spearman correlation coefficient, 0.166) and was associated with recurrent ischemic stroke (*p* = 0.018; Yes, 10.93 ± 16.44 mg/L; No, 6.70 ± 8.13 mg/L); hypertension (*p* = 0.001; Yes, 10.22 ± 14.95 mg/L; No, 6.02 ± 7.21 mg/L); NIHSS score at admission (*p* < 0.001; NIHSS ≤ 15, 5.65 ± 6.41 mg/L; NIHSS > 15, 12.36 ± 17.04 mg/L); ESR (*p* < 0.001; Spearman correlation coefficient, 0.402); ALB (*p* < 0.001; Spearman correlation coefficient, −0.326); FIB (*p* < 0.001; Spearman correlation coefficient, 0.505) and D-dimer (*p* < 0.001; Spearman correlation coefficient, 0.386).

### 3.3. Logistic Regression Analyses

A number of independent predictors of poor outcome after acute ischemic stroke were revealed in multivariate logistic regression analyses (Table 4). Multivariate logistic regression analyses showed that poor outcome at discharge was associated with recurrent acute ischemic stroke (OR, 2.115; 95% CI, 1.094–4.087), non-lacunar stroke (OR, 2.943; 95% CI, 1.436–6.032), DBIL (OR, 1.795; 95% CI, 1.311–2.458), and CRP (OR, 4.890; 95% CI, 3.063–7.808). The associations with age, residence, coronary heart disease, NIHSS score at admission, time from onset of stroke to admission, TBIL, ALB, FIB and D-dimer were not statistically significant, and therefore were not included in the final parsimonious regression model.

## 4. Discussion

In China, stroke is the second most common cause of death and the leading cause of adult disability [2]. Functional disorder in stroke patients remains a huge burden on public health. Exploring the factors influencing functional outcome in stroke patients is critical for risk assessment and interventions aiming to reduce this burden.

To our knowledge, few of the previous studies investigated the relationship between CRP levels and the outcome at discharge. This present study showed that recurrent acute ischemic stroke, non-lacunar stroke, DBIL and high CRP were risk factors of poor outcome at discharge after adjusting for confounding factors. Our findings provide new etiological factors as well as practical implications for stroke patients.

CRP is an acute phase reactant and a marker of inflammation in the serum, as well as the risk of stroke [17,18,19]. It has been suggested that the relationship between increased serum CRP levels and stroke risk is because of the inflammation seen in atherosclerosis.

Many investigators have demonstrated that a high CRP level after ischemic stroke is a predictive factor of poor outcome [7,24,25,26,27,36]. Therefore, testing the CRP level would not only identify patients being at high risk, but would also provide an opportunity for intervention. Similarly, we found that a high CRP level was related to poor outcome at discharge even after adjustment for other significant covariables. A recently published prospective case-control study also reported that an elevated CRP level at admission was an independent predictor of short-term (within 30 days) functional outcome following acute ischemic stroke in Nigerians [25]. However, another study has shown no association between high CRP level at admission and poor outcome [36]. The lack of association in their study might be attributed to the short outcome period (only seven days). In this study, a high CRP level was demonstrated as an important predictor of poor discharge outcome.

In our study, the prognosis of patients with recurrent acute ischemic stroke is worse than that of first-ever stroke. Recurrent acute ischemic stroke is more likely to have poor discharge outcome due to severe disability or death. Anxin Wang et al. [51] found that recurrent stroke after discharge has a relatively large impact on poor functional outcome, as in the study of Erdur et al. [52].

Zhao et al. [44] showed that the incidence of territory infarction was significantly higher than that of non-lacunar infarction in abnormal CRP serum levels, and they exhibited a significant difference in stroke severity. In addition, a prior study [5] showed that an increased level of inflammatory markers may suggest the severity of lesions at perforating arteries and was therefore a potential predictor of lacunar infarction outcome. Furthermore, Dijk et al. [53] reported that an elevated CRP level was likely to be related to cerebral white matter lesions and lacunar infarcts. In our study, lacunar stroke was not associated with the serum CRP levels, which might partly be related to the group of CRP level that was a continuous variable in our study. However, our research confirmed that non-lacunar stroke was another important factor associated with poor discharge outcome in the acute phase.

A novel finding from our study is the significant association between DBIL and poor discharge outcome in the acute phase. A previous study has reported increased bilirubin levels during ischemic stroke, and an association with the severity of symptoms [54]. Xin Li et al. [55] showed that the serum direct bilirubin level was an independent predictor of stroke. The clinical implications of these findings are unclear at present. It remains to be seen whether DBIL is a marker of stroke severity, or is a response to stroke, or a mixture of both.

### Strengths and Weaknesses of the Study

To the best of our knowledge, we studied the relationship between CRP and discharge outcome for the first time. In order to prove the independent association between CRP level and poor outcome, we considered various factors, including demographic data, clinical data, and blood sampling. We strictly selected the inclusion and exclusion criteria to obtain homogenous data and to avoid potential confounding factors. However, we did not exclude pneumonia and lower urinary and antibiotic therapy for the subjects after stroke, which could affect the outcome. Furthermore, different from previous studies [30,36] and clinical experience, age and stroke severity measured by the NIHSS at admission were not associated with poor outcome in our study; the main cause could be the relatively small sample sizes. In addition, the number of poor discharge outcome was relatively small (*n* = 78), because of 13 factors used in a multivariate model. Finally, the CRP level was measured only once at admission. We believe that repeated estimation during hospitalization could be more useful. Therefore, further in-depth studies with larger sample sizes will be warranted to confirm our findings.

## 5. Conclusions

In conclusion, the CRP level measured at admission was found to be an independent predictor of poor outcome at discharge. Other factors, including recurrent acute ischemic stroke, non-lacunar stroke, and DBIL, were also associated with poor outcome at discharge. Further investigations are needed to explore the mechanisms behind this association.

## Figures and Tables

**Table 1:** Comparison of baseline demographic characteristics between poor outcome and good outcome at discharge.

Variable	Good Outcome (MRS ≤ 2) (*n* = 223)	Poor Outcome (MRS > 2) (*n* = 78)	Total	t/χ^2^	*p*
Mean age ± SD, year	63.08 ± 10.44	67.13 ± 10.27	64.13 ± 10.53	−2.961 ^a^	**0.003**
Gender				0.901 ^b^	0.342
Male	131 (58.7%)	41 (52.6%)	172 (57.1%)		
Female	92 (41.3%)	37 (47.4%)	129 (42.9%)		
Residence				7.228 ^b^	**0.007**
Rural	95 (42.6%)	47 (60.3%)	142 (47.2%)		
Urban	128 (57.4%)	31 (39.7%)	159 (52.8%)		
Working				0.448 ^b^	0.503
Yes	72 (32.3%)	22 (28.2%)	94 (31.2%)		
No	151 (67.7%)	56 (71.8%)	207 (68.8%)		
Marital status				0.349 ^b^	0.554
Single	18 (8.1%)	8 (10.3%)	26 (8.6%)		
Married	205 (91.9%)	70 (89.7%)	275 (91.4%)		
Smoking				3.799 ^b^	0.051
Yes	78 (35.0%)	37 (47.4%)	115 (38.2%)		
No	145 (65.0%)	41 (52.6%)	186 (61.8%)		
Alcohol drinking				2.575 ^b^	0.109
Yes	41 (18.4%)	21 (26.9%)	62 (20.6%)		
No	182(81.6%)	57 (73.1%)	239 (79.4%)		

Data presented are *n* (%) or mean (±SD); good outcome defined as Modified Rankin Scale (MRS) ≤ 2; Not working included retired and unemployed; The single marital status included unmarried, divorced, and widowed. ^a^ Independent-sample *t*-test; ^b^ χ^2^ Test.

**Table 2:** Clinical factors related to outcome at discharge in acute ischemic stroke patients.

Variable	Good Outcome (MRS ≤ 2) (*n* = 223)	Poor Outcome (MRS > 2) (*n* = 78)	Total	χ^2^/*z*	*p*
Mean HOD ± SD, day	11.45 ± 3.25	12.08 ± 3.67	11.61 ± 3.37	−1.436 ^a^	0.151
Recurrent ischemic stroke				12.854 ^b^	**<0.001**
Yes	85 (38.1%)	48 (61.5%)	133 (44.2%)		
No	138 (61.9%)	30 (38.5%)	168 (55.8%)		
Comorbid conditions					
Hypertension				0.024 ^b^	0.876
Yes	135 (60.5%)	48 (61.5%)	183 (60.8%)		
No	88 (39.5%)	30 (38.5%)	118 (39.2%)		
Diabetes mellitus (DM)				0.432 ^b^	0.511
Yes	41 (18.4%)	17 (21.8%)	58 (19.3%)		
No	182 (81.6%)	61 (78.2%)	243 (80.7%)		
Coronary heart disease				4.042 ^b^	**0.044**
Yes	64 (28.7%)	32 (41.0%)	96 (31.9%)		
No	159 (71.3%)	46 (59.0%)	205 (68.1%)		
NIHSS score at admission				10.228 ^b^	**0.001**
NIHSS ≤ 15	138 (61.9%)	32 (41.0%)	170 (56.5%)		
NIHSS > 15	85 (38.1%)	46 (59.0%)	131 (43.5%)		
Lacunar stroke				7.685 ^b^	**0.006**
Yes	109 (48.9%)	24 (30.8%)	133 (44.2%)		
No	114 (51.1%)	54 (69.2%)	168 (55.8%)		
Time from onset of stroke to admission (h)				6.323 ^b^	**0.042**
Time ≤ 12	89 (39.9%)	32 (41.0%)	121 (40.2%)		
12 < time ≤ 24	37 (16.6%)	22 (28.2%)	59 (19.6%)		
Time > 24	97 (43.5%)	24 (30.8%)	121 (40.2%)		

Data presented are *n* (%) or mean ± SD; good outcome defined as Modified Rankin Scale (MRS) ≤ 2; HOD, hospital day; NIHSS, National Institute of Health Stroke Scale. ^a^ Non-parametric test; ^b^ χ^2^ Test.

**Table 3:** Characteristics of clinical blood biochemical indexes linked to outcome at discharge in acute ischemic stroke patients.

Variable Mean ± SD	Good Outcome (MRS ≤ 2) (*n* = 223)	Poor Outcome (MRS > 2) (*n* = 78)	Total	t/z	*P*
Routine blood					
CRP (mg/L)	4.97 ± 6.35	18.86 ± 19.05	8.57 ± 12.65	−8.892 ^a^	**<0.001**
WBC (10^9^/L)	7.42 ± 2.15	7.82 ± 2.64	7.52 ± 2.29	−1.332 ^b^	0.184
RBC (10^12^/L)	4.45 ± 0.50	4.40 ± 0.60	4.44 ± 0.53	0.783 ^b^	0.434
HGB (g/L)	137.49 ± 18.86	137.60 ± 15.23	137.52 ± 17.96	−0.046 ^b^	0.963
Biochemical Items					
ESR (mm/h)	19.99 ± 13.27	23.37 ± 16.51	20.87 ± 14.23	−1.815 ^b^	0.071
TBIL (umol/L)	13.51 ± 5.22	15.41 ± 5.75	14.00 ± 5.42	−2.701 ^b^	**0.007**
DBIL (umol/L)	4.25 ± 1.81	5.34 ± 1.78	4.53 ± 1.86	−4.579 ^b^	**<0.001**
ALB (g/L)	40.17 ± 2.91	38.45 ± 7.04	39.73 ± 4.42	−5.738 ^a^	**<0.001**
CHOL (mmol/L)	4.71 ± 1.03	4.63 ± 1.12	4.69 ± 1.05	0.510 ^b^	0.610
TG (mmol/L)	1.67 ± 1.28	1.31 ± 0.60	1.57 ± 1.15	−1.893 ^a^	0.058
HDL (mmol/L)	1.12 ± 0.39	1.12 ± 0.25	1.12 ± 0.36	0.023 ^b^	0.982
LDL (mmol/L)	3.03 ± 0.91	2.91 ± 1.10	3.00 ± 0.96	0.954 ^b^	0.341
GLU(mmol/L)	6.48 ± 2.56	6.54 ± 1.96	6.50 ± 2.41	−0.197 ^b^	0.844
HCY (umol/L)	20.20 ± 13.64	23.51 ± 12.82	21.06 ± 13.49	−1.869 ^b^	0.063
Blood Coagulation					
FIB (mg/dL)	307.41 ± 81.53	346.60 ± 91.46	317.56 ± 85.80	−3.538 ^b^	**<0.001**
D-dimer (ng/mL)	442.40 ± 534.64	930.35 ± 954.95	568.85 ± 701.03	−5.916 ^a^	**<0.001**

Good outcome defined as Modified Rankin Scale (MRS) ≤ 2; CRP, C-reactive protein; WBC, white blood cell; RBC, red blood cell; HGB, hemoglobin; ESR, erythrocyte sedimentation rate; TBIL, total bilirubin; DBIL, direct bilirubin; ALB, albumin; CHOL, total cholesterol; TG, triglyceride; HDL, high density lipoprotein; LDL, low density lipoprotein; GLU, glucose; HCY, homocysteine; FIB, fibrinogen. ^a^ Non-parametric test; ^b^ Independent-sample *t*-test.

**Table 4:** Multivariate logistic regression analysis of significant risk factors for outcome at discharge in acute ischemic stroke patients.

Variable	OR *	95% CI	*p*
Recurrent ischemic stroke			
Yes	2.115	1.094–4.087	**0.026**
No	reference		
Lacunar stroke			
Yes	reference		
No	2.943	1.436–6.032	**0.003**
DBIL (per 1 SD increase)	1.795	1.311–2.458	**<0.001**
CRP (per 1 SD increase)	4.890	3.063–7.808	**<0.001**

OR, Odds ratio; CI, confidence interval; CRP, C-reactive protein; DBIL, direct bilirubin. * Adjusting for age, residence, recurrent ischemic stroke, coronary heart disease, NIHSS score at admission, lacunar stroke, time from onset of stroke to admission, CRP, TBIL, DBIL, ALB, FIB and D-dimer.
